# Pathogenicity and transmissibility of Mpox virus in African dormice

**DOI:** 10.1128/spectrum.01926-25

**Published:** 2026-01-06

**Authors:** Zhaoliang Chen, Lei Zhang, Linzhi Li, Mingjie Shao, Zongzheng Zhao, Chao Shang, Zirui Liu, Juxiang Liu, Yan Liu, Xiao Li, Zhendong Guo

**Affiliations:** 1 State Key Laboratory of Pathogen and Biosecurity, Changchun Veterinary Research Institute, Chinese Academy of Agricultural Sciences 595703 , Changchun, Jilin, China; 2 College of Veterinary Medicine, Hebei Agricultural University 74562, Baoding, Hebei, China; 3 School of Life Science and Engineering, Southwest University of Science and Technology 91609, Mianyang, Sichuan, China; 4 College of Life Sciences, Jilin Agricultural University 85112, Changchun, Jilin, China; National Chung Hsing University, Taichung, Taiwan, Province of China

**Keywords:** Mpox virus, pathogenicity, contact transmission, airborne transmission, dormice

## Abstract

The global spread of Mpox virus (MPXV) poses a major public health challenge, yet reliable and consistent animal models for MPXV evaluation remain limited, which hinders progress in developing treatment strategies and transmission-blocking technologies. As natural hosts, African dormice (*Graphiurus* spp.) are promising candidates, and several studies have partially examined MPXV pathogenicity in dormice. However, the transmission dynamics and viral shedding kinetics of MPXV in dormice remain largely uncharacterized. This study systematically evaluated MPXV pathogenicity and transmissibility in dormice. Experimental results demonstrated that dormice are highly susceptible to MPXV infection. Following intranasal inoculation, MPXV caused significant weight loss, lethal infections, and multi-organ pathological damage, with robust viral replication in respiratory and liver tissues. Notably, MPXV efficiently transmitted among dormice through direct contact, with one-third of contact-exposed dormice shedding infectious viruses and two-thirds exhibiting seropositivity. Additionally, one-third of dormice exposed to airborne particles exhibited seropositivity; however, no infectious viruses were detected in their respiratory tissues. This suggests that the airborne transmissibility of MPXV among dormice is relatively limited. Furthermore, infected dormice continuously released large quantities of virus-laden aerosols, peaking at 12 dpi (3.78 ± 1.01 × 10^6^ copies/dormouse/hour of respiration). Particle size analysis revealed that viral copies in ≥7 µm coarse particles accounted for >90.9% of exhaled viral aerosols during peak shedding (8–14 dpi). These findings indicate that African dormice are a suitable animal model for assessing MPXV infection, pathogenicity, and transmission. Simultaneously, enhanced surveillance of wild dormouse populations is essential given their potential role in MPXV transmission chains.

**IMPORTANCE:**

The global spread of monkeypox virus (MPXV) clade IIb has highlighted the urgent need for reliable animal models to investigate its biological characteristics. This study established an African dormouse (Graphiurus spp.) model and systematically characterized the pathogenicity and transmissibility of a clade IIb strain (hMPXV/China/GZ8H-01/2023). Intranasal inoculation induced significant weight loss, lethal infections, and multi-organ pathology, with robust viral replication in the respiratory and liver tissues. Direct contact transmission was efficient: 1/3 of contact-exposed dormice shed infectious virus, while 2/3 showed seropositivity. Although airborne exposure resulted in 1/3 seropositivity, no infectious virus was detected, thereby indicating limited airborne transmission. Infected dormice emitted abundant virus-laden aerosols, peaking at 3.78 ± 1.01×10⁶ copies/dormouse/hour at 12 days post-infection (dpi). These findings establish African dormice as effective models for studying MPXV infection, pathogenicity, and transmission. Enhanced surveillance of wild dormouse populations is critical due to their potential role in MPXV transmission chains.

## INTRODUCTION

The Orthopoxvirus Mpox, commonly referred to as Mpox virus (MPXV, which will be used throughout the remaining manuscript), is a member of the Orthopoxvirus genus within the Poxviridae family and shares the same genus as the variola virus. Additionally, it is classified as a zoonotic pathogen (1, 2). MPXV can cause Mpox in humans, with clinical manifestations closely resembling those of smallpox (3). The incubation period for MPXV infection varies among individuals and is followed by a 1–5 day prodromal phase lasting 1 to 5 days. Symptoms during this phase may include fever, chills, diaphoresis, headache, fatigue, back pain, malaise, as well as swelling and tenderness of lymph nodes in the cervical, axillary, and/or inguinal regions. A characteristic clinical feature of Mpox is the development of skin lesions that are similar to those observed in smallpox infections (4, 5). Although adult mortality rates are generally low, they can exceed 10% in children and immunocompromised individuals (6).

The first recorded Mpox case occurred in 1958 in crab-eating macaques and rhesus macaques imported from Singapore to Copenhagen, Denmark, for laboratory use (7). The first human case was reported in 1970 in Zaire (now the Democratic Republic of the Congo). Since then, the virus has progressively spread across Africa and to other regions, with endemic prevalence primarily observed in Central and West African countries. Therefore, MPXV has traditionally been classified into two clades: the Central African (Congo Basin [CB]) clade and the West African (WA) clade, with case fatality rates of 10.6% and 3.6%, respectively (3, 8). Historically, Mpox cases outside Africa were rare, with their occurrence mainly linked to international travel or wildlife trafficking (9–11). However, since May 2022, an increasing number of human Mpox cases have emerged in countries and regions outside the traditional recognized endemic areas. This recent multi-national outbreak exhibits a distinct epidemiological pattern: the majority of confirmed cases are not linked to known exposure to infected individuals or travel to endemic regions. Furthermore, unlike previous outbreaks, the current transmission route is predominantly sexual contact, especially among men who have sex with men (12, 13). According to the U.S. Centers for Disease Control and Prevention (CDC), MPXV can also spread via direct contact with affected areas such as rashes, ulcers, or scabs; contact with contaminated objects such as clothing or bedding; and inhalation of respiratory droplets, aerosols, or oral secretions from an infected individual (14). The World Health Organization declared Mpox a “Public Health Emergency of International Concern” in July 2022 and again in August 2024 (15). As the global spread of the human MPXV accelerates, and considering the adoption of a non-stigmatizing classification, MPXV is now categorized into clades I, IIa, and IIb (16). Clade I corresponds to the prior CB clade; clade IIa corresponds to the traditional WA clade; and clade IIb contains genomes sampled from the 2017–2019 outbreaks and the 2022–present global outbreaks. Due to the closer phenotypic and phylogenetic similarity of the clades IIa and IIb, clades IIa and IIb are collectively referred to as the “WA clade” (16). As MPXV continues to spread, evolve, and mutate, it may be undergoing continuous adaptation to humans (17, 18). Therefore, monitoring the pathogenicity and transmissibility of the currently prevalent clade IIb strain is of critical importance, which contributes to the development of scientific, rigorous, and effective prevention and control strategies.

Appropriate animal models are essential for understanding MPXV’s pathogenesis, transmission routes, and epidemiology. Recent studies have identified several susceptible species as viable models for evaluating pathogenicity, vaccines, and therapeutics (19). These include rodents models such as the African dormouse (20), prairie dog (21), Gambian pouched rat (22), African rope squirrel (23, 24), ground squirrel (25), and rabbit model (26, 27), as well as non-human primate models, including *Macaca mulatta* (28), *Macaca fascicularis* (29), and marmosets (30). Although inbred mouse strains, such as BALB/c and C57BL/6, exhibit relatively low susceptibility to MPXV infection, their distinct immune profiles make them valuable experimental models for mechanistic studies (31). Among these models, the black-tailed prairie dog (*Cynomys ludovicianus*) is one of the few in which MPXV transmissibility has been experimentally characterized, involving direct contact with infected animals, indirect contact with contaminated materials (e.g., bedding), and respiratory transmission routes (32, 33). Studies on transmissibility remain limited across the majority of other animal models. For many species, even basic transmission metrics—such as aerosol spread efficiency and viral shedding duration—have yet to be systematically elucidated. This knowledge gap impedes a comprehensive understanding of MPXV’s host-specific transmission dynamics.

The African dormouse (*Graphiurus* spp.), a small rodent species and natural host of MPXV, is a promising animal model for studying the virus. Prior studies have confirmed its susceptibility to lethal MPXV infection, with multi-organ viral replication and associated pathological damage (20, 34). Its small size, affordability, ease of handling, and adaptability to controlled environments make it ideal for high-throughput studies requiring statistically significant sample sizes. However, research on MPXV in dormice has predominantly focused on pathogenicity**,** with viral transmissibility remaining unexplored. Critical questions remain unanswered. How efficiently does MPXV spread among dormice? What are the dominant transmission routes (e.g., direct contact, aerosols)? What viral shedding kinetics underlie these transmission processes? Addressing these gaps could offer valuable insights into MPXV’s ecological transmission and inform spillover risk assessments.

In this study, we obtained a clade IIb isolate of MPXV (hMPXV/China/GZ8H-01/2023) and comprehensively assessed its pathogenicity and transmissibility in African dormice. We first developed a dormouse model of MPXV infection to systematically characterize the pathogenic profile of the virus in this rodent species, including clinical manifestations, tissue tropism, and histopathological changes. Transmissibility assessments were subsequently performed via direct contact and aerosol transmission routes to quantify transmission efficiency and delineate the epidemiological potential of these routes. Additionally, we conducted time-series analyses of viral aerosol shedding from infected dormice, providing critical insights into environmental viral dissemination. This study demonstrates that African dormice serve as a reliable animal model for MPXV research, enhancing understanding of MPXV epidemiology and informing public health strategies to mitigate zoonotic spillover and spread.

## MATERIALS AND METHODS

### Virus and cell

The hMPXV/China/GZ8H-01/2023 strain of MPXV (GenBank: PP778666.1) was kindly provided by the Eighth Affiliated Hospital of Guangzhou Medical University and propagated in Vero E6 cells. This strain, isolated from a patient in Guangzhou, China, in 2023, is classified as clade IIb (16). The master seed virus obtained after proliferation is stored at −80°C. Vero E6 cells were cultured in a humidified incubator at 37°C with 5% CO_2_. The culture medium comprised Dulbecco’s modified Eagle medium (DMEM, Sigma-Aldrich) supplemented with 10% fetal bovine serum (FBS; Invitrogen), 50 U/mL penicillin, and 50 µg/mL streptomycin.

### Animals and viral challenge

Male African dormice (*Graphiurus* spp.), aged 10–12 months, and weighing 20–30 g, were used in this study. The animals were reared under controlled laboratory conditions at the Changchun Veterinary Research Institute, maintained at 24 ± 2°C with 40%–70% relative humidity and a 12 h light/dark. Additionally, food and water were provided *ad libitum*. Animals were randomly assigned to experimental groups and acclimatized in the ABSL-3 facility for 2–3 days before the start of the experiment. For viral challenge experiments, animals were anesthetized via isoflurane inhalation and, following confirmation of anesthesia, were intranasally inoculated with 50 µL of MPXV solution containing 10^5.3^ TCID_50_ (10^5.15^ PFU).

### Pathogenicity of MPXV in dormice

**Fig 1:**
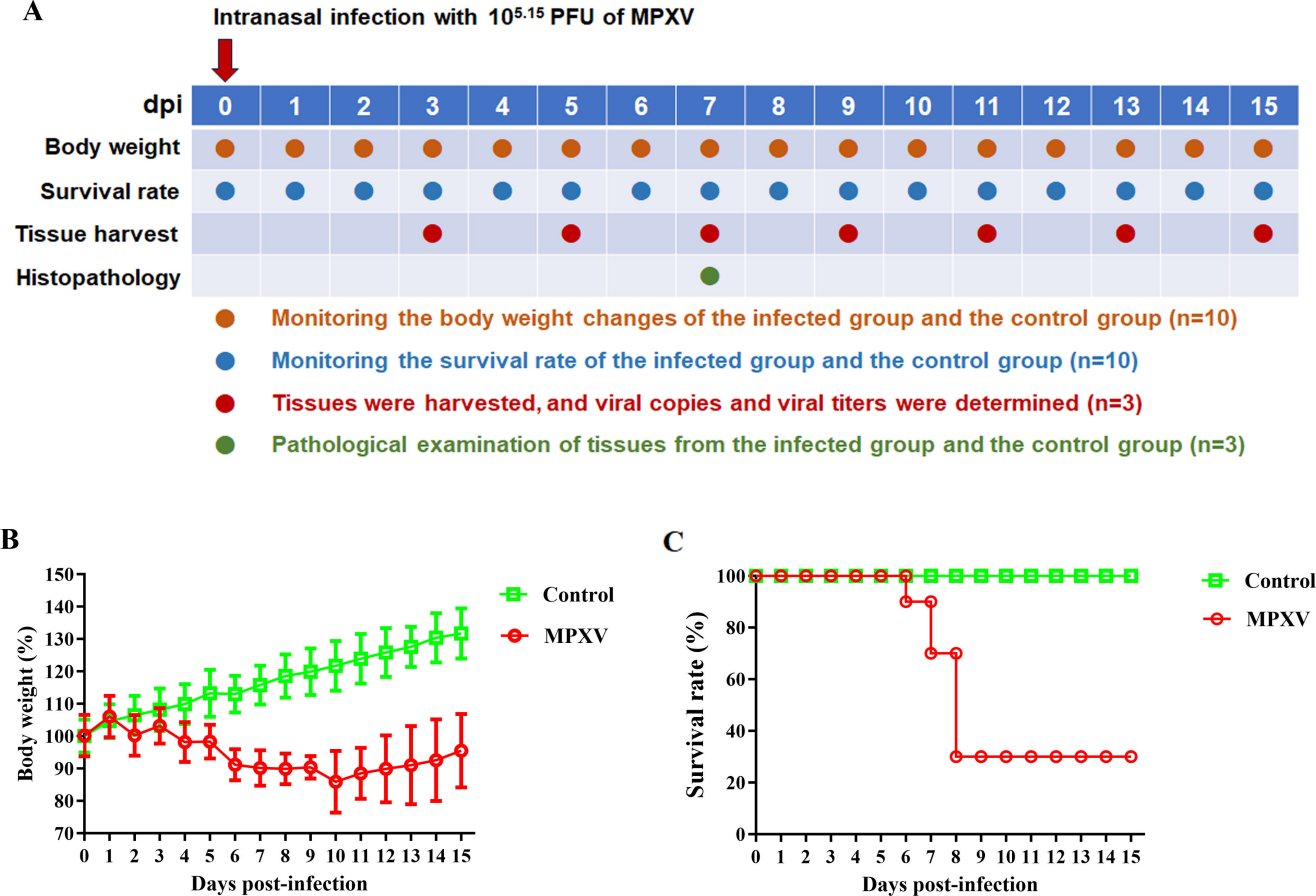
Body weight and survival rate in dormice infected with Mpox virus. Ten healthy male dormice (10–12 months old) were intranasally inoculated with 10^5.3^ TCID_50_ (10^5.15^ PFU) of Mpox virus (MPXV) to establish the infected group. A separate group of 10 animals received an equivalent volume of DMEM as a control. Body weight changes and survival rates in both groups were monitored daily over a 15-day period. (**A**) Experimental flowchart for evaluating MPXV pathogenicity in dormice. (**B**) Relative body weight. (**C**) Survival rate. dpi, days post-infection.

The experimental flowchart for evaluating MPXV pathogenicity in dormice is shown in Fig. 1A.

To monitor body weight changes and survival rate of dormice following MPXV infection, 20 dormice were randomly assigned to two groups: infected (*n* = 10) and control (*n* = 10). The infected group was intranasally inoculated with 10^5.3^ TCID_50_ (10^5.15^ PFU) of MPXV, while the control group was intranasally inoculated with an equal volume of DMEM. Body weight and survival of these dormice were monitored daily for 15 days post-challenge, and dormice that lost more than 25% of their initial body weight were humanely euthanized.

To determine the tissue distribution of MPXV and assess pathological damage in dormice following MPXV infection, 80 dormice were inoculated intranasally with 10^5.3^ TCID_50_ (10^5.15^ PFU) of MPXV, and three control dormice received DMEM. On 3, 5, 7, 9, 11, 13, and 15 days post-infection (dpi), tissues from three randomly selected infected dormice were collected, including nasal turbinate, brain, heart, lung, liver, trachea, kidney, spleen, intestine, and testis. These tissues were subsequently placed in 1 mL PBS with 2% penicillin-streptomycin, homogenized, centrifuged, and the supernatant was collected. The supernatant was diluted and inoculated into Vero-E6 cells, and the TCID_50_ was determined using the Reed-Muench method. Due to the high mortality rate in dormice inoculated with 10^5.3^ TCID_50_ (10^5.15^ PFU) of MPXV (Fig. 1), a larger initial cohort (*n* = 80) was used to ensure sufficient animals for sampling across seven time points.

On 7 dpi, tissues from three infected and three control dormice were fixed in paraformaldehyde, embedded in paraffin, sectioned, and stained with hematoxylin and eosin (H&E) for histopathological analysis.

### Transmissibility of MPXV in dormice

To evaluate the transmissibility of MPXV between dormice via direct contact and airborne transmission, 48 donor dormice were intranasally inoculated with 10^5.3^ TCID_50_ (10^5.15^ PFU) of MPXV. At 24 hpi, 24 donor dormice were transferred to four clean cages and co-housed with 24 naïve recipient dormice for direct contact transmission studies. The animals were randomly assigned to four cages, with six donor dormice housed with six naïve direct-contact recipient dormice in each cage. Additionally, the remaining 24 donor dormice were transferred to four wire-frame cages adjacent to 24 naïve recipient dormice for airborne transmission studies. The animals were also randomly assigned to four cages, with six donor dormice housed adjacent to six naïve airborne-exposure recipient dormice in each cage. The donor dormice and airborne-exposure recipient dormice were separated by a double-layered wire mesh barrier with a 2 cm gap, which prevented direct contact while allowing respiratory transmission.

On 7, 10, 13, and 16 days post-infection or exposure, the nasal turbinate, trachea, and lungs were collected from six donor or six recipient dormice in each group and homogenized in 1 mL PBS supplemented with 2% penicillin-streptomycin. TCID_50_ was then determined, and viral nucleic acid was quantified using quantitative real-time PCR. On 16 days post-infection or exposure, whole blood was collected from dormice in each group and incubated overnight at 4°C for serum separation. The serum samples were heat-inactivated at 56°C for 30 min to eliminate complement activity. Samples were then serially diluted and mixed 1:1 with viral inoculum containing 100 TCID_50_. The mixtures were incubated in 96-well plates for 1 h at 37°C before the addition of Vero-E6 cells. Cells were cultured under conditions promoting adherence and growth at 37°C. Four days later, the neutralizing antibody titer in serum was determined by evaluating the number of wells with cytopathic effects (CPE) and the number of wells showing complete antibody-mediated protection across each serum dilution.

### Collection of exhaled viral aerosols from dormice

A total of 20 dormice were intranasally inoculated with 10^5.3^ TCID_50_ (10^5.15^ PFU) of MPXV. The larger cohort (*n* = 20) was used due to high mortality rate observed following infection (Fig. 1). Exhaled breath samples from a fixed subset of four dormice were collected on 2, 4, 6, 8, 10, 12, 14, and 16 dpi using six-stage Andersen impactors (TE-10-800; Tisch Inc., Cleves, OH, USA). If any of the designated four dormice died or met euthanasia criteria during the study, they were replaced with surviving individuals from the original cohort to ensure continuous sampling with a consistent group of four animals at each time point. Collection parameters were set at a flow rate of 28.3 liters per minute for 60 min per session. Six-stage Andersen impactors classify aerosol particles in the exhaled breath into six size ranges (>7 µm, 4.7–7 µm, 3.3–4.7 µm, 2.1–3.3 µm, 1.1–2.1 µm, and 0.65–1.1 µm) based on aerodynamic diameter. Aerosol particles within each size range were collected onto separate, pre-sterilized gelatin filters (Sartorius, Germany), as previously described (35). The aerosol samples were then divided into two equal portions: one for quantitative detection of viral nucleic acid, and the other for viral titer determination using the TCID_50_ method.

### Determination of the median infective dose (ID_50_)

Twenty dormice were randomly assigned to four groups, with five animals per group. Each group was intranasally inoculated with MPXV at doses of 10^4.3^ TCID_50_, 10^3.3^ TCID_50_, 10^2.3^ TCID_50_, and 10^1.3^ TCID_50_, respectively. On 7 dpi, the dormice were euthanized, and respiratory tissues, including the nasal turbinates, trachea, and lungs, were collected to determine viral titers. The ID_50_ for intranasally administered MPXV was calculated using probit analysis based on infection rates in each group.

### Viral nucleic acid detection

DNA was extracted using the TIANamp Virus DNA/RNA Kit (TIANGEN, China) and quantified by quantitative real-time PCR (Q-RT-PCR) with the One Step PrimeScript RT-PCR Kit (TaKaRa, Japan), following the manufacturers’ protocols. The primers and probe targeting the *F3L* gene of MPXV were as follows: Forward: 5′-CTCATTGATTTTTCGCGGGATA-3′; Reverse: 5′-GACGATACTCCTCCTCGTTGGT-3′; Probe: 5′ FAM-CATCAGAATCTGTAGGCCGT-3′ TAMRA. A standard plasmid containing the *F3L* gene was used to generate a standard curve for absolute quantification of viral copy numbers. Q-RT-PCR was performed on an ABI 7500 System (ThermoFisher, Waltham, MA, USA).

### Statistical analysis

Data were analyzed using GraphPad Prism software (San Diego, CA, USA). One-way analysis of variance (ANOVA) was used to determine statistically significant differences between groups. *P* < 0.05 was considered statistically significant. All assays were performed in triplicate and are representative of at least three independent experiments.

## RESULTS

### Body weight changes and survival rate of dormice following MPXV infection

We evaluated the pathogenicity of MPXV in dormice. The experimental flowchart is presented in Fig. 1A. Following intranasal inoculation with MPXV, the animals showed a significant decrease in body weight, with a peak reduction of 14.1% compared to their initial weight on 10 dpi (Fig. 1B). Peak mortality occurred between 6 and 8 dpi, resulting in a final mortality rate of 70% (Fig. 1C). In contrast, body weight of dormice in the control group increased steadily, and no deaths occurred during the experiment (Fig. 1).

### Tissue distribution of MPXV in dormice

**Fig 2:**
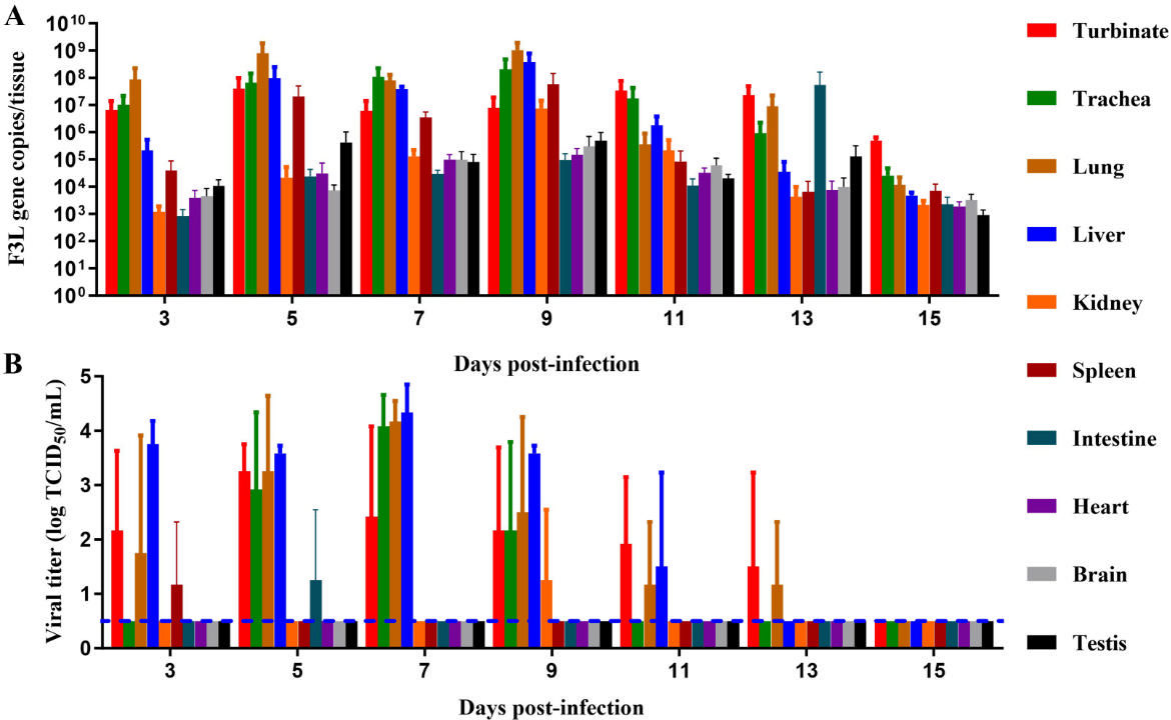
Tissue distribution of MPXV in dormice. Eighty male dormice were intranasally inoculated with 10^5.3^ TCID_50_ (10^5.15^ PFU) of MPXV. Three animals per time point were humanely euthanized on 3, 5, 7, 9, 11, 13, and 15 days post-infection. The larger cohort (*n* = 80) ensured that at least three animals per time point were available for tissue collection, accounting for the high mortality rate observed in dormice inoculated with 10^5.3^ TCID_50_ of MPXV (Fig. 1C). The nasal turbinates, trachea, lungs, liver, kidneys, spleen, intestine, heart, brain, and testes were collected and homogenized in PBS. After centrifugation, supernatants were collected for analysis. Viral nucleic acid quantity (**A**) and viral titer (**B**) were subsequently quantified. Each color-coded column represents a distinct organ. Data are presented as mean ± SD. Dashed lines indicate the lower limit of detection.

We conducted a comprehensive investigation into the dispersion and replication kinetics of MPXV in dormouse. Following intranasal infection, the virus rapidly spread to multiple organs. Nucleic acid testing confirmed the presence of viral nucleic acids in all tested organs of the infected dormice, suggesting that the virus may disseminate systemically via the bloodstream to all tested organs (Fig. 2A). Infectious viruses were robustly detected in the nasal turbinates, trachea, lungs, and liver, with peak titers on 7 dpi (Fig. 2B). In contrast, infectious viruses appeared sporadically in the kidneys, spleen, and intestines, while none was detected in the heart, brain, or testes throughout the study.

### MPXV infection-induced pathological tissue damage in dormice

**Fig 3:**
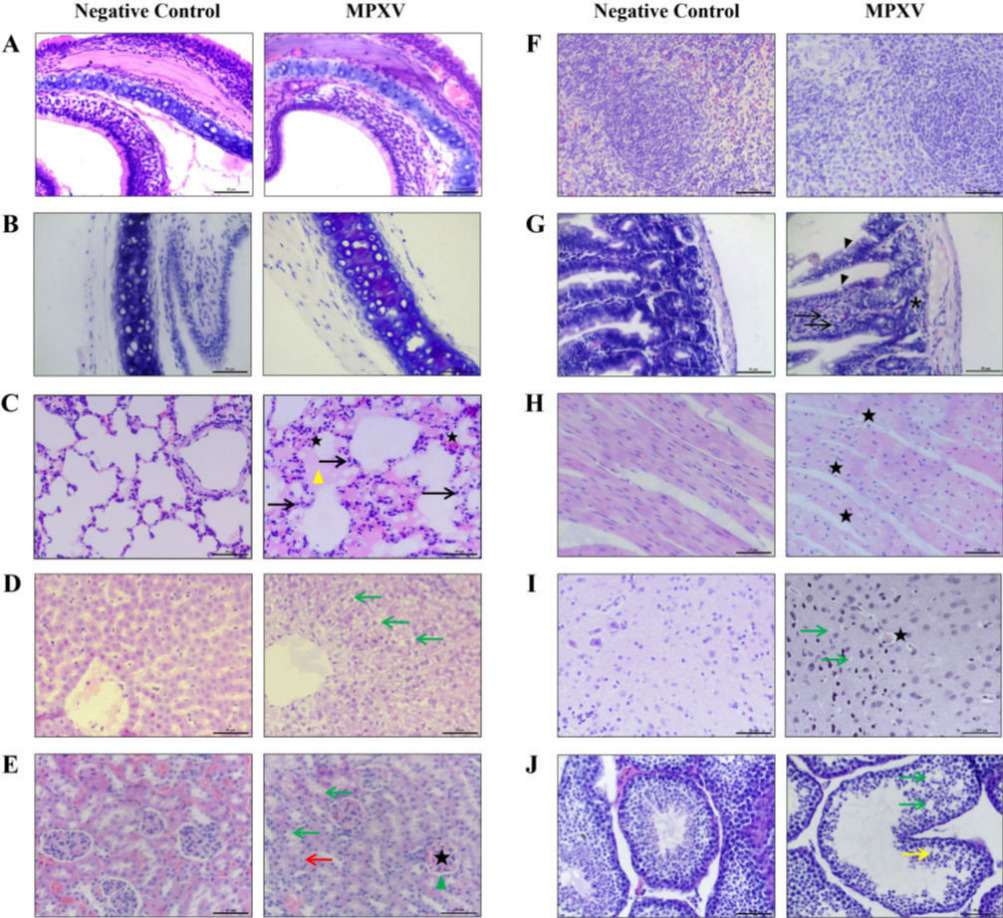
Histopathological analysis of multi-organ damage following Mpox virus (MPXV) infection. Male dormice were intranasally inoculated with either 10^5.3^ TCID_50_ (10^5.15^ PFU) of MPXV or an equal volume of DMEM, serving as the infected and control groups, respectively. On the 7 dpi, multiple organs were collected for analysis, including (**A**) nasal turbinate, (**B**) trachea, (**C**) lungs, (**D**) liver, (**E**) kidneys, (**F**) spleen, (**G**) small intestine, (**H**) heart, (**I**) brain, and (**J**) testes. Tissues were fixed in formalin, embedded in paraffin, and stained with hematoxylin-eosin (H&E) for pathological examination. Symbols: pentagram—vascular or capillary hyperemia (including glomerular vascular hyperemia and dilation); black arrows—inflammatory cell infiltration; yellow triangles—serous exudates; green arrows—cytoplasmic vacuolization or presence of vacuoles between cells; red arrows—cell granular degeneration; green triangle—narrowing of Bowman’s capsule cavity; black triangles—increased number of goblet cells; asterisks—reduction of small intestinal glands; yellow arrow—reduced spermatids in seminiferous tubules. Scale: 50 µm or 100 µm.

We further investigated histopathological changes in dormice following MPXV infection (Fig. 3). In the infection group, only the nasal turbinate (Fig. 3A), trachea (Fig. 3B), and spleen (Fig. 3F) showed no significant pathological damage. Other organs, including the lungs, liver, kidneys, intestine, heart, brain, and testis, displayed varying degrees of pathological injury. In contrast, no obvious pathological changes were observed in tissues of the negative control group.

Figure 3C shows lung. In the control group, lung tissue appeared normal and intact. In contrast, in the infected group, the lung interstitium was markedly widened, and alveolar walls showed varying degrees of thickening. Pulmonary interstitial capillaries exhibited congestion (adjacent to the pentagram), accompanied by inflammatory cell infiltration (lymphocytes, black arrows) and serous exudates within the alveolar spaces (yellow triangles).

Figure 3D shows liver. In the control group, the structures of hepatocytes, hepatic cords, and hepatic sinusoids were relatively well-preserved. In contrast, the infected group showed significant vacuolar degeneration of hepatocytes (green arrow), hepatocyte swelling, and marked narrowing of hepatic sinusoids.

Figure 3E shows kidney. In the control group, renal corpuscles displayed normal morphology, with no histopathological changes in renal tubular epithelial cells. Compared to the control, the infected group showed mild hyperemia and swelling of renal glomeruli (pentacle), narrowing of Bowman’s capsule cavity (green triangle), granular degeneration of renal tubular epithelial cells (red arrow), and cytoplasmic vacuolization (green arrow).

Figure 3G shows intestine. In the control group, the intestinal villi were intact, and the mucosa, submucosa, and muscularis layers exhibited a well-defined stratified structure. Compared to the control, the infected group showed a notable increase in goblet cells (black triangles) within the intestinal mucosa, accompanied by slight thickening of the muscularis layer, lymphocytic infiltration within the lamina propria (black arrow), and reduced small intestinal glands (asterisk).

Figure 3H shows heart. In the control group, the myocardium exhibited a uniformly consistent color, with myocardial fibers arranged orderly and a distinct, wide interstitial space between myocytes and muscle fibers. In contrast, the infected group showed markedly uneven myocardial coloration, disorganized arrangement of myocardial fibers, significant myocyte swelling, and congested capillaries (adjacent to the pentagram).

Figure 3I shows brain. In the control group, the brain exhibited normal cerebral cortex structure and healthy neurons. In contrast, the infected group showed capillary congestion in the cerebral cortex (pentagrams), and neuronal degeneration was evident, characterized by pyknosis or loss of nuclei and cytoplasmic vacuolization (green arrows).

Figure 3J shows testis. In the control group, spermatogenic cells at all levels within the seminiferous epithelium of the seminiferous tubules were clearly and orderly arranged. In the infected group, the number of spermatogenic cells in the seminiferous epithelium decreased, and intercellular vacuoles (green arrows) appeared within the seminiferous epithelium. Compared with the control group, the structure of the seminiferous epithelium was disorganized, with evident severe exfoliation of spermatogenic cells. A marked reduction in sperm count within the seminiferous tubules was observed (yellow arrow).

### Direct contact and airborne transmissibility of MPXV in dormice

**Fig 4:**
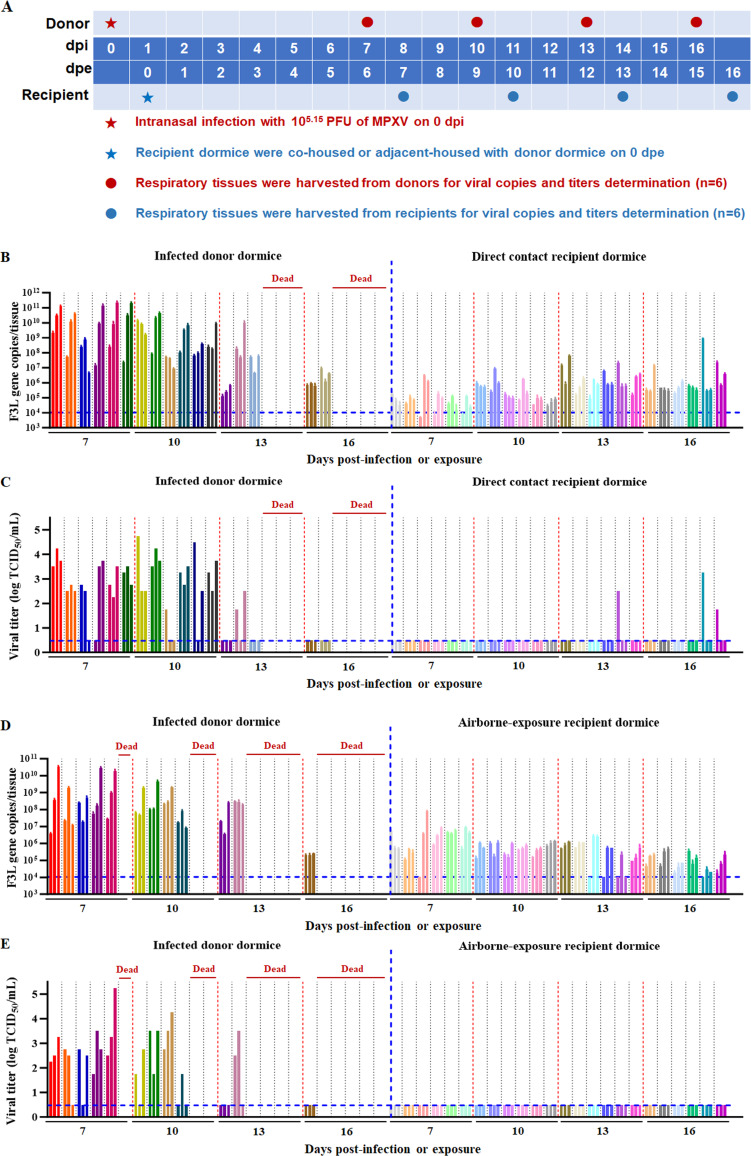
Direct contact and airborne transmissibility of MPXV in dormice. (**A**) Experimental flowchart for evaluating MPXV transmissibility in dormice. For direct contact transmission, infected donor dormice were co-housed with direct contact recipient dormice, allowing direct physical contact. For airborne transmission, donor animals were housed in a wire-frame cage adjacent to airborne exposure recipient dormice, separated by a double-layered wire mesh barrier, with a 2 cm gap, preventing direct contact but allowing respiratory transmission. Donors were intranasally inoculated with 10^5.3^ TCID_50_ (10^5.15^ PFU) of MPXV. At 24 hpi, recipient dormice were co-housed (direct contact) or adjacent-housed (airborne exposure) with donors. On 7, 10, 13, and 16 days post-infection or exposure, six donor or recipient dormice per group were humanely euthanized. Nasal turbinates, trachea, and lungs were collected to quantify viral nucleic acid copies and titers. The insets show viral nucleic acid concentrations (**B**) and viral titers (**C**) in respiratory tissues of dormice from the direct contact transmission experiment, as well as viral nucleic acid concentrations (**D**) and viral titers (**E**) in the respiratory tissues of dormice from the airborne transmission experiment. In each graph (**B–E**), the left panel depicts the animals that were inoculated with MPXV through the intranasal route (donor animals), and the right panel illustrates the animals that were subsequently exposed naturally through either direct contact or airborne transmission (recipient animals). Each set of three same-colored column bars represents the three respiratory tissues (from left to right: nasal turbinate, trachea, and lungs) from an individual dormouse. Dashed lines indicate the lower limit of detection.

We performed a comprehensive evaluation of MPXV transmissibility among dormice via direct contact and airborne routes (Fig. 4A). We successfully detected viral nucleic acid in respiratory tissues of all animals in the direct contact group (Fig. 4B), demonstrating that MPXV particles can be transmitted between dormice through direct contact. On 13 days post-exposure (dpe), infectious viruses were detected in one of six recipient dormice (1/6) (Fig. 4C). Additionally, on 16 dpe, infectious viruses were detected in one-third of the recipient dormice (2/6), and seroconversion occurred in two-thirds of the animals (4/6; Fig. S1A). These findings indicate that MPXV can transmit through direct contact among dormice. Moreover, infectious viruses in recipient dormice were found only in the nasal turbinates, suggesting that infection likely occurred via the nasal cavity.

We also successfully detected viral nucleic acid in the respiratory tissues of all animals in the airborne transmission group (Fig. 4D), indicating that viral particles can be transmitted via the airborne route. However, no infectious virus was detected in this group during the observation period (Fig. 4E). On 16 dpe, one-third of dormice became seropositive (2/6; Fig. S1B). These results suggest that although MPXV particles can be airborne, they are insufficient to induce substantial infection. Taken together, MPXV exhibits limited airborne transmissibility among dormice.

### Concentrations and particle size distribution of viral aerosols exhaled by MPXV-infected dormice

**Fig 5:**
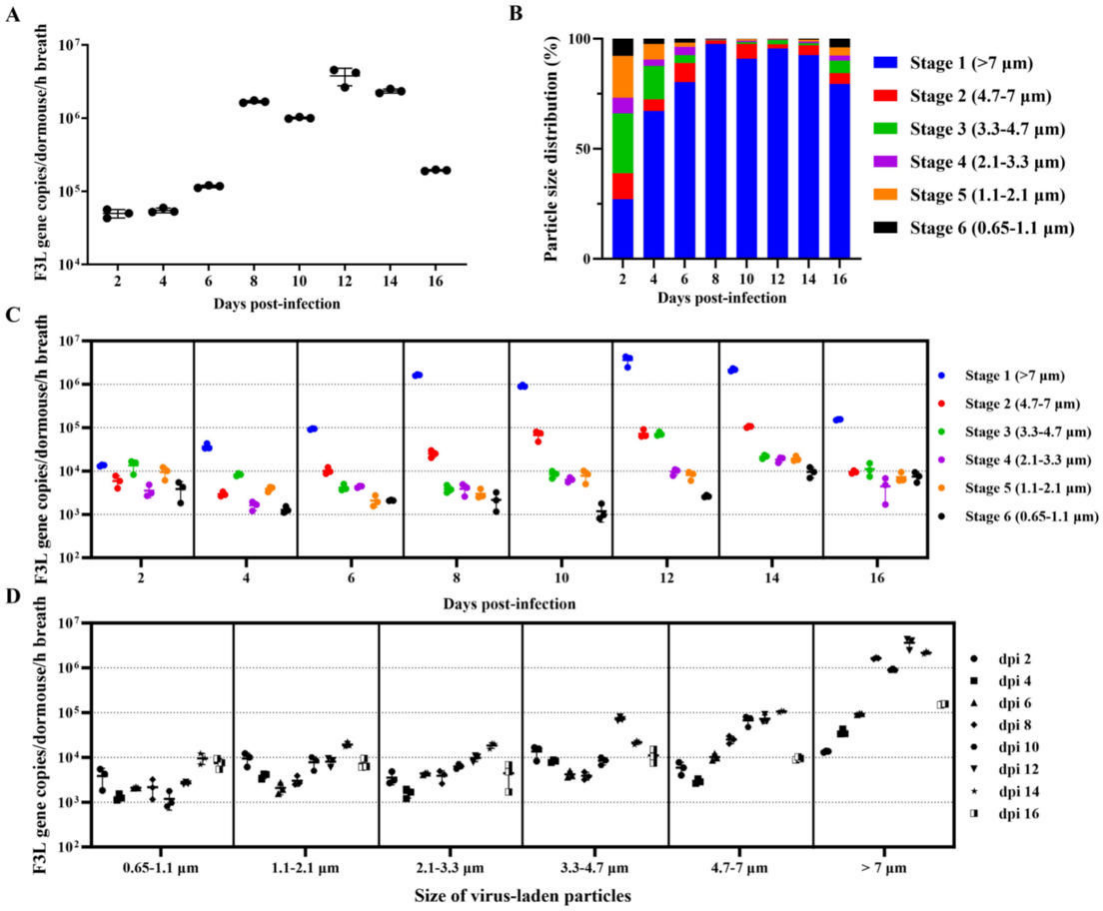
Concentration and particle size distribution of virus-laden aerosols exhaled by MPXV-infected dormice. Twenty dormice were intranasally inoculated with 10^5.3^ TCID_50_ (10^5.15^ PFU) of MPXV. A larger cohort (*n* = 20) was used due to high mortality rate observed following infection (Fig. 1C). From 2 to 16 dpi, a six-stage Andersen impactor was used to collect virus-laden aerosols exhaled by a fixed subset of four dormice on alternate days. If any of the designated four dormice died or met euthanasia criteria, they were replaced with surviving animals from the initial cohort to ensure continuous sampling with a consistent group of four animals at each time point. (**A**) Total concentrations of virus-laden aerosols exhaled by infected dormice. (**B**) Size distribution of virus-laden particles in the exhaled viral aerosols. (**C**) Quantity and size distribution of virus-laden particles exhaled by infected dormice, categorized by particle size range. (**D**) Quantity and size distribution of virus-laden particles exhaled by infected dormice, categorized by post-infection time points. Data in panels **A**, **C**, and **D** are shown as mean ± SD.

In the airborne transmission experiment, we successfully detected viral nucleic acid in all airborne exposure recipient dormice, demonstrating that infected dormice can release viral aerosols. Furthermore, viral particles in exhaled aerosols were capable of transmitting between dormice and inducing seroconversion in one-third of exposed dormice. Therefore, we further analyzed the viral shedding dynamics in exhaled aerosols of MPXV-infected dormice. A six-stage Andersen impactor was used to collect aerosols exhaled by infected dormice on alternate days from 2 to 16 dpi. The total concentration of viral particles in exhaled aerosols showed an increasing trend, peaking at 12 dpi (3.78 ± 1.01 × 10^6^ viral copies per dormouse per hour’s breath), followed by a gradual decrease by 16 dpi (Fig. 5A).

We further investigated the aerodynamic diameter distribution of virus-laden particles in exhaled aerosols. Our observations revealed that exhaled aerosols contained a significantly higher abundance of larger-diameter particles compared to smaller ones (Fig. 5B). During the peak phase of viral aerosol shedding (8–14 dpi), the majority of virus-laden particles were ≥7 µm in diameter, accounting for over 90.9% of the total size distribution. Particles ranging from 0.65 to 7 µm constituted only a minor fraction of the distribution.

Figure 5C and D provide a more detailed analysis of the quantity and size distribution of virus-laden particles in exhaled aerosols. The quantity of virus-laden particles larger than 7 µm remained consistently at approximately 10^6^ to 10^7^ copies per dormouse per hour’s breath during the middle and late stages of infection (8 to 14 dpi). This was followed by particles in the 4.7 to 7 µm range, which were maintained at approximately 10^5^ copies per dormouse per hour’s breath. The concentration of virus-laden particles with a <4.7 µm diameter remained consistently low throughout the infection period, rarely exceeding 10^4^ copies per dormouse per hour’s breath. As time progressed, the concentration of virus-laden particles in exhaled aerosols increased predominantly in coarse particles ≥4.7 µm in diameter, while the quantity of fine particles ranging from 0.65 to 4.7 µm exhibited relatively minor fluctuations (Fig. 5D).

### Median infective dose (ID_50_) of MPXV for intranasal infection in dormice

**Fig 6:**
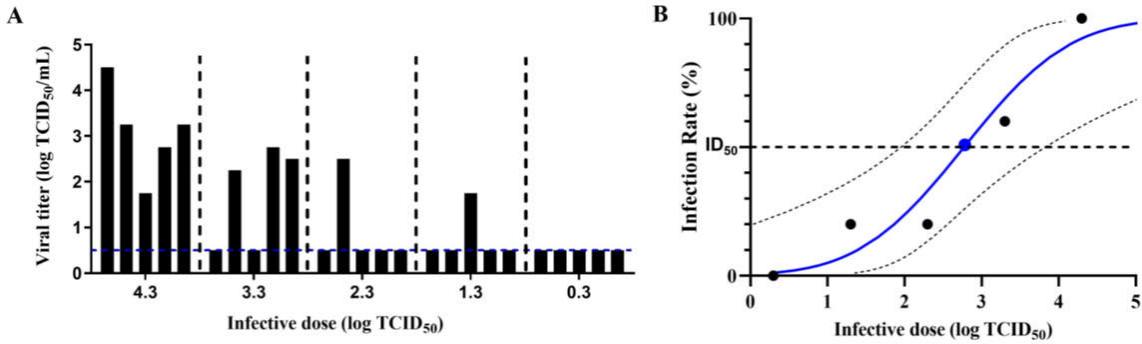
Median infective dose (ID_50_) of MPXV via nasal instillation in dormice. (**A**) Groups of five dormice were intranasally inoculated with serial dilutions of MPXV 10^4.3^ TCID_50_, 10^3.3^ TCID_50_, 10^2.3^ TCID_50_, and 10^1.3^ TCID_50_. At 7 dpi, the dormice were euthanized, and their respiratory tissues, including the nasal turbinates, trachea, and lungs, were collected to determine viral titers. Each bar represents the maximum titer detected in the respiratory tissues of an individual dormouse. Dashed line indicates the lower detection limit. (**B**) Probit analysis was used to calculate the ID_50_ for MPXV. Dashed lines represent the 95% confidence interval.

Finally, we conducted an in-depth investigation into the susceptibility of dormice to MPXV via respiratory tract infection by intranasally administering varying doses of MPXV, from low to high, to groups of five dormice each. The maximum viral titer in the respiratory system (nasal turbinates, trachea, and lungs) of each dormouse is shown in Fig. 6A, while infection rates for each group are summarized in Table 1. Both the infection rate and viral titer in the respiratory system followed a dose-dependent pattern. At doses below 10^2.3^ TCID_50_, the infection rate remained relatively low. However, at 10^3.3^ TCID_50_, the infection rate increased significantly, and at 10^4.3^ TCID_50_, it approached 100%. Probit analysis was conducted to estimate the ID_50_ of MPXV infection in dormice (Fig. 6B). The ID_50_ for MPXV infection in dormice was determined to be 10^2.773^ TCID_50_, with a 95% confidence interval ranging from 10^1.958^ to 10^3.827^ TCID_50_.

**TABLE 1:** Infection rates of dormice inoculated with varying doses of MPXV

Infective dose (lg TCID_50_)	Number of infected dormice	Total dormice population	Infection rate (%)
0.3	0	5	0
1.3	1	5	20
2.3	1	5	20
3.3	3	5	60
4.3	5	5	100

## DISCUSSION

MPXV was originally endemic to Central and West Africa but has recently spread rapidly worldwide, raising major public health concerns. Understanding the pathogenicity and transmissibility of the currently prevalent clade IIb strain is critical for effective scientific prevention and control of the Mpox epidemic. In this study, we established an African dormouse (*Graphiurus* spp.) model and systematically characterized the pathogenicity and transmissibility of a clade IIb strain (hMPXV/China/GZ8H-01/2023) isolated from patients in Guangzhou, China. Song et al. used the same strain as our study and evaluated its pathogenicity in Spanish dormice (*Dryomys nitedula*) (36). We found the clade IIb strain exhibited consistent pathogenic features in both dormouse species, including similar clinical signs (weight loss and fatal infection), viral tissue tropism (presence of infectious viruses in respiratory tissues and liver), and pulmonary pathology (interstitial widening, inflammatory cell infiltration, and alveolar exudation). Minor differences included 70% vs 80% mortality rate in African vs Spanish dormice at comparable inoculation doses, and slight variations in organ tropism (efficient replication in the spleen of Spanish dormice but reduced replication in African dormice). These consistent pathogenic characteristics across dormouse species support the use of dormice as reliable animal models for MPXV research. Earl et al. and Schultz et al. used the same dormouse species as our study and assessed the pathogenicity of a clade I strain (20, 34). Compared to the clade I strain, our clade IIb isolate showed lower mortality rate in dormice (Fig. 1C) but a similar viral tissue distribution (Fig. 2). Histopathological damage caused by clade IIb MPXV was less severe, with no evident lesions in the nasal turbinate or spleen (Fig. 3), while the clade I strain caused nasal mucosal inflammation and lymphatic necrosis in the spleen (34), suggesting that the clade IIb strain is comparatively less virulent. Previous studies in humans and non-human primates have also shown that clade I is generally more pathogenic than clade IIa (8, 37). These divergent findings further underscore the differences in pathogenicity among MPXV clades.

In the viral transmission study, infectious viruses were detected in three recipient dormice within the contact transmission group, whereas none were identified in the airborne transmission group (Fig. 4). These findings suggest that MPXV is efficiently transmitted among dormice through direct contact, while its airborne transmissibility in this species remains relatively limited. It is worth noting that although the direct contact transmission experiment design is commonly used in the contact transmissibility evaluation of various viruses (38–40), it has a methodological limitation: co-housed animals share the same microenvironment preventing clear differentiation between transmission via direct physical contact and airborne droplets within the cage. Therefore, the observed transmission outcomes likely reflect a combination of both routes. However, in scenarios involving direct physical contact (e.g., nasal/oral contact, grooming), recipient animals are expected to be exposed to larger quantities of infectious secretions (e.g., nasal discharge, saliva) compared to airborne particles, which are more prone to dilution, inactivation, and exist at relatively lower concentrations (41–43). Higher viral loads acquired through direct contact with infectious secretions are more likely to overcome host defenses and initiate infection. Therefore, contact-based transmission likely plays a more significant role in this setup. While infectious virus was detected in the nasal turbinates of contact recipient dormice, this only indicates the nasal mucosa as a primary infection site and does not differentiate between exposure to secretions (contact) and aerosols (airborne). In contrast, the airborne transmission setup used strict physical separation with double wire mesh spaced 2 cm apart, eliminating direct contact and limiting transmission to airborne particles. The absence of infectious virus in recipient dormice from the airborne transmission group provides direct evidence that airborne particles alone are insufficient to mediate transmission in this model. Consequently, physical contact—not airborne transmission—is the primary driver of MPXV transmission among dormice.

Previous research on MPXV transmissibility across animal populations remains limited to studies involving black-tailed prairie dogs. Hutson et al. conducted a contact transmission experiment among black-tailed prairie dogs using a clade IIa strain (MPXV-USA-2003-044) (32). Viable virus was detected in the upper respiratory tract of recipient prairie dogs as early as 13 dpe. A similar result was observed in our dormouse experiments with the clade IIb strain, where infectious virus was isolated from the nasal turbinates of recipient dormice at the same time point (Fig. 4C), indicating the upper respiratory tract as the primary portal for contact infection. Regarding airborne transmission, Hutson et al. found inefficient respiratory spread among prairie dogs, with airborne transmission efficiencies of 16.7% and 0% for clades I and IIa strains, respectively—contrasting the more efficient direct contact transmission (33). Our dormouse experiments, using similar methods, confirmed that the clade IIb strain also showed limited airborne transmissibility (Fig. 4D and E). Collectively, these findings across both species emphasize that contact transmission is more efficient than airborne transmission.

MPXV transmission characteristics in dormice closely resemble those observed in humans, as both transmission routes are primarily dependent on close contact. Epidemiological surveillance data demonstrate that human-to-human transmission of MPXV relies heavily on direct contact (44). Among unvaccinated individuals, the secondary attack rate ranged from 7.5% to 12.3% among household contacts, while dropping to 3.3% among non-household close contacts (45, 46). Moreover, the transmission chain rarely extends beyond secondary cases; tertiary and quaternary events (infections stemming from secondary and tertiary cases, respectively) are only sporadically reported (47, 48). The restricted airborne transmissibility of MPXV observed in dormice (Fig. 4) aligns with human populations: person-to-person airborne transmission remains unconfirmed, with only limited indirect evidence supporting its potential (49, 50). However, this possibility cannot be entirely excluded (51, 52). Further studies, including more human case data and airborne transmission experiments across additional animal models, are essential for a comprehensive understanding.

Two potential factors may limit the airborne transmissibility of MPXV among dormice. First, the 50% infectious dose (ID_50_) for respiratory tract infection with MPXV is relatively high compared to other respiratory pathogens. Our study determined that the ID_50_ of MPXV in dormice via intranasal infection was approximately 10^2.7^ TCID_50_ (Fig. 6), whereas respiratory pathogens like influenza virus and SARS-CoV-2 typically exhibit ID_50_ values around 10^1^ TCID_50_ (53). The higher ID_50_ helps explain MPXV’s limited airborne transmissibility within dormouse populations. Second, the particle size of airborne pathogens plays a critical role in airborne transmission efficiency (54). Droplets larger than 4.7 µm travel shorter distances, while smaller particles (<4.7 µm) remain airborne longer, disperse more widely, and penetrate deeper into the respiratory tract. Previous studies by Zaucha et al. (55) and Barnewall et al. (56) developed MPXV inhalation models in cynomolgus monkeys using clade I strain (Zaire V79-I-005) aerosols with median particle sizes of 1.2 µm and 1.08–1.15 µm, respectively. These small particles can reach the alveoli and induce infection. In contrast, MPXV-laden aerosols exhaled by infected dormice in our study had a median size exceeding 7 µm (Fig. 5). These larger particles travel only short distances and only tend to deposit in the upper respiratory tract, reducing respiratory transmission efficiency in dormice.

Our study confirms that African dormice are susceptible to MPXV and can transmit the virus to conspecifics through contact. As key players in MPXV’s natural transmission cycle, African rodents—including dormice—serve as primary natural hosts, supporting viral persistence and spread within ecosystems (57). In African regions, frequent human infections from contact with virus-carrying rodents underscore their epidemiological relevance. The ecological traits of these rodents—including their wide-ranging activity (spanning multiple habitats such as forests, farmlands, and human settlements), tendency to forage near human dwellings (increasing opportunities for human-rodent contact), omnivorous feeding habits (potentially facilitating cross-species interactions), and high reproductive rates (sustaining stable population sizes)—facilitate the cross-regional and cross-species viral transmission (44). Specifically, wild African dormice may contribute to MPXV circulation in two ways: as hosts, they replicate and shed virus over extended periods, sustaining environmental presence; as vectors, infected individuals transmit the virus to cohabiting rodents and non-human primates during routine activities, amplifying ecosystem-wide circulation and increasing zoonotic spillover risks. Additionally, dormice kept as pets may represent a critical link in MPXV transmission via international trade, potentially posing a significant public health threat (58).

In summary, the African dormouse is a suitable animal model for assessing MPXV infection, pathogenicity, and transmission dynamics. Dormice infected with MPXV display distinct pathogenic features, and the virus can be transmitted between populations via direct contact. Although aerosol transmission is relatively limited capability, experimental evidence shows that infected dormice can exhale substantial viral aerosols, posing a potential public health risk. Simultaneously, intensified surveillance of wild dormouse populations is essential given their possible role in MPXV transmission chains. Finally, it is recommended to develop and implement regulatory frameworks to effectively disrupt potential animal-to-human transmission. These findings provide critical evidence for strengthening Mpox prevention and control strategies.

## Data Availability

The data will be made available upon request.

## SUPPLEMENTAL MATERIAL

Fig. S1 spectrum.01926-25-s0001.docx Serum neutralizing antibody titers in dormice on day 16 post-inoculation or -exposure.

ASM does not own the copyrights to Supplemental Material that may be linked to, or accessed through, an article. The authors have granted ASM a non-exclusive, world-wide license to publish the Supplemental Material files. Please contact the corresponding author directly for reuse.
