# Supplementary material for: Pathogenicity and transmissibility of Mpox virus in African dormice
Source: Microbiol Spectr. 2026 Jan 6;14(2):e01926-25. doi: 10.1128/spectrum.01926-25 (PMC12889042; doi:10.1128/spectrum.01926-25)
Supplement: Fig. S1 — Serum neutralizing antibody titers in dormice on day 16 post-inoculation or -exposure. [file spectrum.01926-25-s0001.docx]

**Supplementary Materials**

**Title:** Pathogenicity and transmissibility of Monkeypox virus in dormice

**Figure legends**


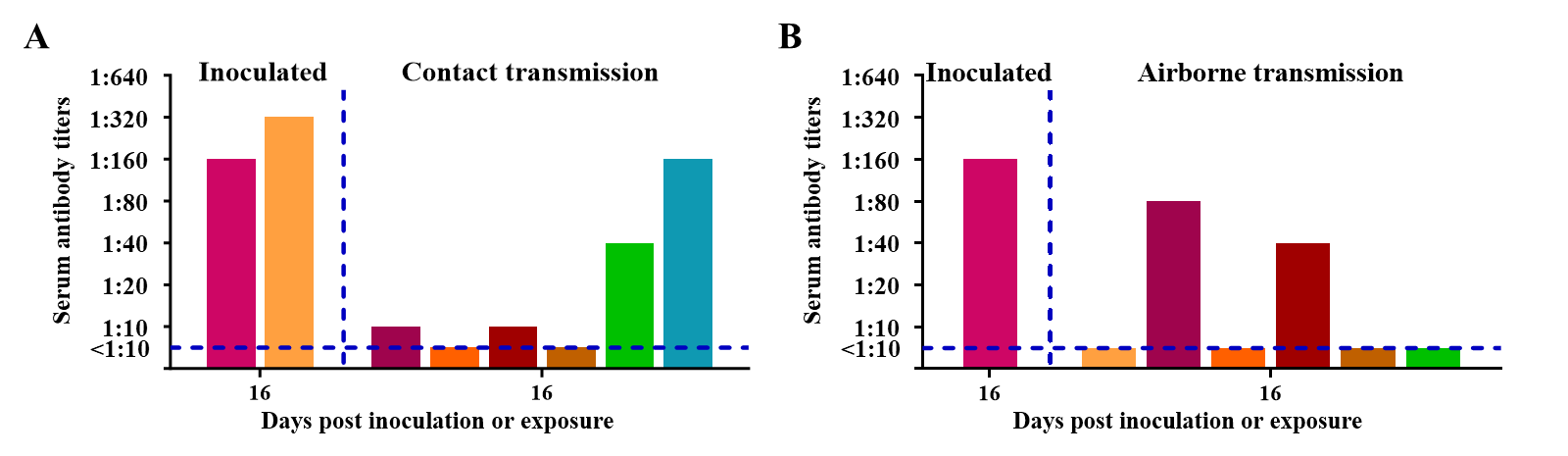


**Supplementary Figure 1.** **Serum neutralizing antibody titers in dormice on day 16 post-inoculation or exposure.** In the experiment assessing direct contact and airborne transmissibility in dormice, whole blood samples were collected from infected or exposed dormice on day 16 post-inoculation or exposure, and serum neutralizing antibody titers were subsequently measured in VeroE6 cells. The results were presented as the highest antibody dilution capable of fully protecting Vero-E6 cells from cytopathic effects (CPE). The differently colored data bars within each group represent individual animals. The number of data bars reflects the number of animals that survived on day 16 post-inoculation or exposure, while the dashed line indicates the lower limit of detection.
